# Rapid Assessment of Insect Pest Outbreak Using Drones: A Case Study with *Spodoptera exigua* (Hübner) (Lepidoptera: Noctuidae) in Soybean Fields

**DOI:** 10.3390/insects14060555

**Published:** 2023-06-15

**Authors:** Yong-Lak Park, Kushal Naharki, Roghaiyeh Karimzadeh, Bo Yoon Seo, Gwan-Seok Lee

**Affiliations:** 1 Entomology Program, Division of Plant and Soil Sciences, West Virginia University, Morgantown, WV 26506, USA; kn00019@mix.wvu.edu (K.N.); roghaiyeh.karimzadeh@mail.wvu.edu (R.K.); 2 Department of Plant Protection, Faculty of Agriculture, University of Tabriz, Tabriz 5166614888, Iran; 3 Crop Foundation Division, National Institute of Crop Science, Rural Development Administration, Wanju 55300, Republic of Korea; seoby@korea.kr; 4 Division of Crop Protection, National Institute of Agricultural Sciences, Rural Development Administration, Wanju 55300, Republic of Korea; gslee12@korea.kr

**Keywords:** drone, pest detection, site-specific pest management, UAS, remote sensing, SADIE, NDVI, satellite

## Abstract

**Simple Summary:**

When an insect pest outbreak occurs, it is crucial to quickly assess the damage to manage the outbreak effectively. This study investigated a serious outbreak of the beet armyworm, *Spodoptera exigua* (Hübner) (Lepidoptera: Noctuidae), that occurred in soybean fields in South Korea. We conducted an aerial survey of 31 soybean blocks within the outbreak region using drones. The aerial images were analyzed to quantify soybean defoliation and to investigate the spatial patterns of the soybean damage by *S. exigua*. The results of this study showed that the aerial survey was an effective and rapid method for estimating the defoliation of soybeans caused by *S. exigua*. Moreover, it was found that the aerial survey followed by image analysis was more economical and required less time than a conventional ground survey, especially when the number of target soybean blocks subject to the survey was more than 15 blocks. Overall, the study demonstrated the effectiveness of using an autonomous drone and image analysis to conduct a low-cost aerial survey of soybean damage caused by *S. exigua* during its outbreak.

**Abstract:**

Rapid assessment of crop damage is essential for successful management of insect pest outbreaks. In this study, we investigated the use of an unmanned aircraft system (UAS) and image analyses to assess an outbreak of the beet armyworm, *Spodoptera exigua* (Hübner) (Lepidoptera: Noctuidae), that occurred in soybean fields in South Korea. A rotary-wing UAS was deployed to obtain a series of aerial images over 31 soybean blocks. The images were stitched together to generate composite imagery, followed by image analyses to quantify soybean defoliation. An economic analysis was conducted to compare the cost of the aerial survey with that of a conventional ground survey. The results showed that the aerial survey precisely estimated the defoliation compared to the ground survey, with an estimated defoliation of 78.3% and a range of 22.4–99.8% in the 31 blocks. Moreover, the aerial survey followed by image analyses was found to be more economical than the conventional ground survey when the number of target soybean blocks subject to the survey was more than 15 blocks. Our study clearly demonstrated the effectiveness of using an autonomous UAS and image analysis to conduct a low-cost aerial survey of soybean damage caused by *S. exigua* outbreaks, which can inform decision-making for *S. exigua* management.

## 1. Introduction

Rapid surveys and assessments of insect pest outbreaks are critical for timely management of decision-making and response to the outbreaks. Ground surveys and aerial sketch mapping by experts on small airplanes have traditionally been used for large-scale surveys of pest outbreaks in field crops and forests, respectively. When the damage is extensive, satellite images may be used to assess the outbreak. However, aerial sketch mapping can be prone to subjectivity error, and remote sensing using satellites has low spatial resolution, making it difficult to directly detect and assess pest outbreaks when damage is scattered across a large geographic area. For example, the United States Department of Agriculture (USDA) Forest Service uses a combination of field surveys and digital aerial sketch mapping, but this method can be influenced by factors such as the experience of the sketch mapper, types of signs and symptoms, aircraft type, and flight speed [1]. Remote sensing using satellites has been used for surveying insect pest outbreaks but has limited applicability in operational pest-management programs [2] due to low image resolution. As a result, there is a need for alternative methods such as unmanned aerial systems and image analysis to conduct low-cost and rapid surveys of pest outbreaks, which can inform timely and effective management decisions.

Unmanned aircraft systems (UAS), commonly known as drones, have become an attractive tool for aerial surveys of crop stress and damage caused by pests [3,4]. UAS flights over agricultural lands or forests require fewer logistics and field crew members compared to manned airplanes [5]. Recent advances in image processing and analysis tools, coupled with high-resolution imagery obtained by UAS, provide a unique opportunity for field image processing capability [6]. UAS have several advantages compared to manned airplanes and satellite imagery for monitoring crop stress and damage, including safety, cost, flexibility, and modularity. Unlike satellite imagery, UAS imagery can be acquired in real-time and tailored to meet site-specific survey and management strategies by locating specific areas that require additional monitoring [6]. The modularity of onboard payloads, combined with an onboard control system, allows for easy retrofitting with a wide range of high-resolution imaging payloads. Recent significant advances in miniaturized flight control technology and expanded capabilities of UAS for autonomous flight offer the possibility of conducting aerial surveys repeatedly and even without human intervention [7].

The beet armyworm, *Spodoptera exigua* (Hübner) (Lepidoptera: Noctuidae), is a key pest of vegetables, field crops, and floricultural crops, with over 90 species of plants in 18 families being reported as host plants globally [8,9,10,11]. The damage by *S. exigua* is predominantly caused by larvae that feed on leaves resulting in feeding signs of defoliation. Outbreaks of *S. exigua* could be associated with weather patterns [9,12], its short life cycle (16–42 days from egg to adult), high fecundity (700–1300 eggs per female), number of generations per year [13,14,15], its spatial distribution pattern [16], and large-scale migration of adults [12,17]. In soybean fields, we observed heavy damage by *S. exigua* causing nearly 100% defoliation.

Current recommendations for *S. exigua* control include counting the number of larvae and applying insecticides as needed; the economic threshold for chemical control is 6.3 larvae/plant for cabbage [18]. Chemical control is the most common practice to control *S. exigua* in South Korea, but the strong insecticide resistance of *S. exigua* has made the control of the moth extremely difficult [19,20]. Specifically, while first and second instars are relatively susceptible to insecticides, third to fifth instars are tolerant to the insecticides, and they generally hide within plants and thus lower the chance of exposure to foliar-applied insecticides. Biological control agents including the entomopathogenic fungus *Nomuraea rileyi* [21] and a nuclear polyhedrosis virus (NPV) have been identified [22] but are currently not commercially available. Therefore, early detection of damage by *S. exigua* is considered an ideal preventative control measure. Although early detection of *S. exigua* might be possible by using doppler weather radar when the swarm of *S. exigua* adults migrates during a major outbreak [23,24], the technology is not readily available to growers and pest control service providers.

In July and August of 2018, an outbreak of *S. exigua* occurred in soybean fields (*Glycine max* [L.]) in Jeollabuk-do, the western region of South Korea, covering an area of 1701 ha near the cities of Kimje, Gunsan, and Iksan. An emergency ground survey was conducted by the government agencies of South Korea to estimate the soybean damage caused by *S. exigua* and to make management decisions such as applying pesticides or reseeding soybean or alternative crops. Taking advantage of the outbreak, we conducted an aerial survey with UAS simultaneously with the ground survey. Our hypothesis was that the aerial survey of *S. exigua* damage using UAS and image analysis could be more efficient than typical ground-based visual surveys. The objectives of this study were (1) to develop a protocol for a rapid assessment of *S. exigua* outbreaks in soybean fields using rotary-wing UAS and image analysis, (2) to compare the efficiency of aerial surveys with conventional ground surveys, (3) to determine the spatial pattern of *S. exigua* damage using spatial statistics, and (4) to check the applicability of satellite imagery for detecting soybean damage caused by *S. exigua* at a regional scale.

## 2. Materials and Methods

### 2.1. S. exigua Outbreak and Survey Site

**Figure 1:**
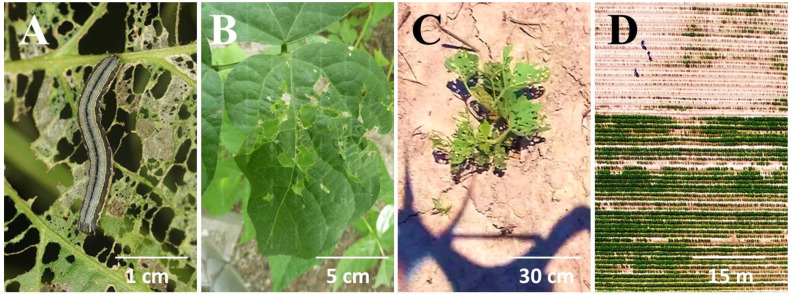
Damage of soybean caused by *S. exigua* from the ground (**A**,**B**) and aerial (**C**,**D**) views. (**A**), a larva feeding on soybean; (**B**), a typical sign of soybean damage caused by *S. exigua*; (**C**,**D**), aerial views of soybean damage at 5 and 50 m above the ground, respectively.

This study was conducted in Juksan Township (N 35.759012, W 126.789840) close to the city of Kimje where the most serious outbreak of *S. exigua* was reported during the 2018 outbreak in South Korea. An initial field visit by the entomologists at the National Institute of Agricultural Sciences of South Korea confirmed that the larvae feeding on soybean foliage were exclusively *S. exigua* (Figure 1).

### 2.2. Ground Survey and Damage Assessment

A team of 19 field-survey experts from interagency organizations, including the National Institute of Agricultural Sciences, Province Agricultural Research and Extension Station, and Agricultural Extension and Technology Center, conducted a conventional ground survey to estimate the damage caused by *S. exigua* in the study site for a rapid response to the outbreak. The survey involved selecting 30–50 soybean plants in each soybean block and rating the damage using five categories: severe (75–100% of soybean damaged), high (50–75%), medium (25–50%), low damage (1–25%), and no damage (0%). The location of soybean blocks surveyed, time taken by 19 surveyors, and damage estimation were recorded.

### 2.3. Aerial Survey and Damage Assessment

**Figure 2:**
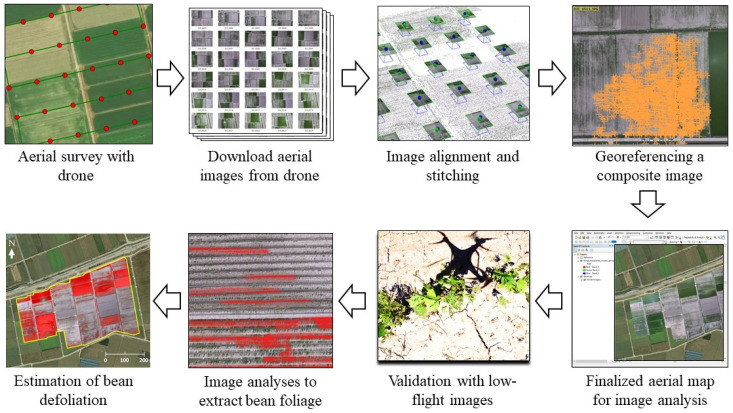
UAS operation and image analysis protocol used in this study. Each step above was automated by autopilot for UAS flight and macro or batch processing for image analysis.

A three-step protocol for assessing damage in soybean fields caused by *S. exigua* was developed with the goal of efficiently completing an aerial survey (Figure 2). The first step involved deploying a rotary-wing UAS (DJI Phantom 3 Advanced; SZ DJI Technology Co., Ltd., Shenzhen, China) to capture aerial imagery. The second step involved downloading the aerial images and stitching them together to generate a geo-referenced composite image of the study site. In the third step, image analyses were conducted to estimate the amount of damage caused by *S. exigua*.

**Figure 3:**
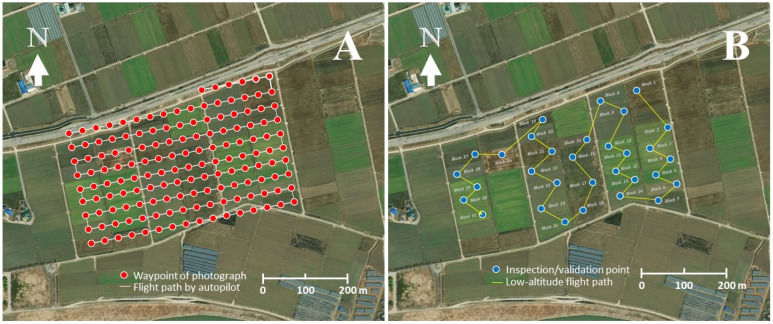
Two different UAS flight plans for rapid assessment of soybean damage caused by *S. exigua*. (**A**), UAS flight path for an autopilot operation at 50 m above the ground; (**B**), a low-altitude flight path to confirm soybean defoliation by *S. exigua* at 5–10 m above the ground.

The UAS was equipped with an RGB camera capable of both photography and videography at a resolution of 1800 p. During the UAS flight, a series of aerial photographs were taken with 80% image overlap between two consecutive images taken. Two different aerial survey methods were employed: (1) a high-altitude survey in autonomous flight mode conducted at 50 m above the ground, and (2) a low-altitude spatially targeted survey conducted at 5–10 m above the ground to confirm *S. exigua* damage on individual soybean plants in each block (Figure 3).

### 2.4. Image Processing and Analysis

Following the completion of the flight missions, the aerial images were downloaded from the UAS. For the high-altitude survey, the images were aligned according to the pre-planned flight path (Figure 3A) and processed using Pix4DMapper software (Pix4D, Prilly, Switzerland) to generate a geo-referenced composite image. The composite image was then ortho-mosaicked with ArcInfo^®^ 10 software (ESRI, Redland, CA, USA) to confer spatial attributes to the map coordinate system. Before conducting the image analysis to estimate the damage caused by *S. exigua*, a validation survey using a low-altitude flight was performed to confirm that the damage was indeed caused by defoliation.

To assess the extent of defoliation caused by *S. exigua* in each soybean block, ArcInfo and Photoshop CS4 (Adobe Inc., San Diego, CA, USA) were used to analyze the composite image. The green pixels in the image were counted to estimate the amount of soybean foliage that remained after *S. exigua* feeding. This method was calibrated by comparing the number of pixels counted in an undamaged area and by calculating the percent reduction due to defoliation based on the number of green pixels present in the damaged area.

### 2.5. Determining the Efficiency of the Aerial Survey with UAS

To determine the efficiency of the aerial survey with UAS, a cost analysis was conducted. Based on flight information recorded in the UAS, we extracted the time of UAS operation for both high- and low-altitude aerial surveys (T_UAS_). Additionally, time for image processing and analysis (T_analysis_), the number of soybean blocks surveyed by UAS flights (N_block_), the expert labor fee per hour for the UAS pilot and image analyst (F_expert_), and the technology fee per hour (F_technology_) were recorded. We set USD 50 per hour for an expert fee and USD 100 for F_technology_ based on average commercial rental rates (i.e., USD 50 for UAS rental and USD 50 for software rental). The total cost for an aerial survey with UAS (C_aerial_) per soybean block was calculated as:

C_aerial_ = [(T_UAS_ + T_analysis_) × (F_expert_ + F_technology_)]/N_block_

To compare the aerial survey with the conventional ground survey, we calculated three variables associated with the ground survey: the number of soybean blocks to survey (N_block_), the time required for the ground survey (T_ground_), and the hourly labor fee (F_labor_) of USD 10 per field surveyor. The total cost per block by a conventional ground survey (Conventional) per block was calculated as:

C_conventional_ = (F_labor_ × T_ground_)/N_block_

Regression analyses were conducted to determine the relationships between the cost and time for different numbers of soybean blocks to survey and those for each of the aerial and ground surveys. All the statistical analyses were conducted with SAS [25] at an error rate of 0.05.

### 2.6. Spatial Patterns of Soybean Damage by S. exigua

We conducted spatial analyses with geostatistics and spatial analysis by distance indices (SADIE) to characterize the spatial pattern and to test the significance of spatial aggregation of soybean damage. UAS images taken at 50 m above the ground were used for the analyses. Soybean blocks with >95% defoliation were excluded from the analyses, as they showed near complete defoliation. The images of soybean defoliation were reclassified into two classes (defoliated or healthy pixels) using ArcGIS Pro 10. Each soybean block was divided into 54-m-by-54-m grids, and the number of pixels representing soybean defoliation in each grid was counted to measure the amount of damage in the grid. The data were analyzed using geostatistics (i.e., semivariogram modeling) to quantify the degree of the spatial dependence and to characterize the spatial structure [26] of soybean damage. Geostatistical analyses were conducted using GS+ 10 (Gamma Design Software, Plainwell, MI, USA). The best theoretical semivariogram models were selected based on minimum residual sums of squares (RSS) and maximum r^2^ values. Three semivariogram parameters were used to describe the spatial structure of soybean damage by *S. exigua*: range, sill, and nugget [27]. Range is the distance at which the semivariance reaches a maximum and represents the lag distance beyond which samples are spatially independent. The sill is the value of the semivariance at any distance greater than or equal to the range. The nugget is the value of the semivariance when lag distance equals zero. By using the these parameters, the degree of spatial dependence (*DD*) was calculated with the following formula:

$$DD=\left(\frac{C}{C_{0}+C}\right)\times 100$$

where *C* is sill and *C*_0_ is nugget. Spatial dependency is considered weak, moderate, and strong when *DD* is ≤25%, 26–75%, and ≥76%, respectively [28].

Although semivariograms can quantify spatial dependency, they do not determine spatial clustering or aggregation. Therefore, SADIE was used to test the statistical significance of spatial aggregation or clustering of soybean damage by *S. exigua* under the null hypothesis of complete randomness [29]. The spatial pattern was determined by calculating the index of aggregation (*I*_a_), and the statistical significance of aggregation was determined with associated probability (*P*_a_). A value of *I*_a_ = 1 indicates a random spatial distribution, *I*_a_ > 1 reveals an aggregated pattern, and *I*_a_ < 1 is indicative of a regular or uniform spatial distribution. The aggregation was considered significant when *P*_a_ < 0.05. Overall spatial clustering was tested by calculating mean clustering indices ($\overset{-}{v_{i}}$ and $\overset{-}{v_{j}}$) and their associated probabilities, *P*$\overset{-}{v_{i}}$ and *P*$\overset{-}{v_{j}}$. A value of *P*$\overset{-}{v_{i}}$ < 0.05 confirmed the presence of significant spatial clustering into patches and *P*$\overset{-}{v_{j}}$ < 0.05 indicated a high level of spatial clustering into gaps [30]. These analyses were conducted by using SADIEShell version 2.0 (Rothamsted Experimental Station, Harpenden Herts, United Kingdom).

### 2.7. Regional-Scale Soybean Damage by S. exigua

To identify soybean fields damaged by *S. exigua* in the outbreak region of South Korea, we analyzed time series satellite data for change detection. We utilized the normalized difference vegetation index (NDVI) before, during, and after the *S. exigua* outbreak. The NDVI is a measure that detects and quantifies the presence of live green vegetation by analyzing reflected light in the visible and near-infrared (NIR) bands. Consequently, NDVI values can serve as indicators of the health or condition of vegetation within each pixel of a satellite image.

We used Landsat 8 Operational Land Imager/Thermal Infrared Sensor (OLI/TIRS) Level 2 imagery with a spatial resolution of 30 m to calculate NDVI values before, during, and after the *S. exigua* outbreak within the study site region. Specifically, we downloaded Landsat 8 OLI/TIRS Level 2 imagery with 11 spectral bands from the U.S. Geological Survey Earth Explorer for Path 115, Row 035. The NDVI values were calculated using the equation: NDVI = (NIR − Red)/(NIR + Red), where Red represents band 4 and NIR represents band 5.

To identify regions affected by *S. exigua* damage, we subtracted the NDVI values during the damage from those before the damage using a raster calculator. Similarly, we subtracted the NDVI values during the damage from those after the damage to locate regions where regrowth or replanted crops occurred within the damaged area.

## 3. Results

### 3.1. Ground Survey and Damage Assessment

**Figure 4:**
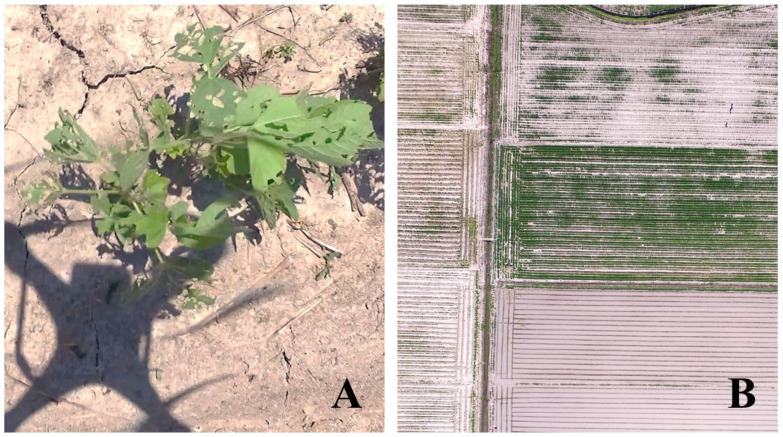
Example aerial survey images at two different altitudes: a low-altitude flight at 5–10 m above the ground for validation aerial survey to confirm the presence of defoliation (**A**) and a high-altitude flight for aerial mapping of soybean defoliation by *S. exigua* at 50 m above the ground (**B**).

A ground survey by experts within the outbreak area showed 53.3%, 31.1%, 13.3%, and 2.2% severe, high, medium, and low damage to soybean plants, respectively. The ground survey also found that *S. exigua* larvae feeding on the soybean plants were mostly second and third instars with fewer fourth instars, indicating the damage was in progress, and thus urgent control of *S. exigua* larvae was needed. The surveyors also observed and reported that soybean blocks planted late experienced higher damage, and *S. exigua* larvae had completely defoliated soybean plants, including stems, in some soybean blocks, causing nearly 100% crop loss (Figure 4).

### 3.2. Aerial Survey and Image Analysis

**Figure 5:**
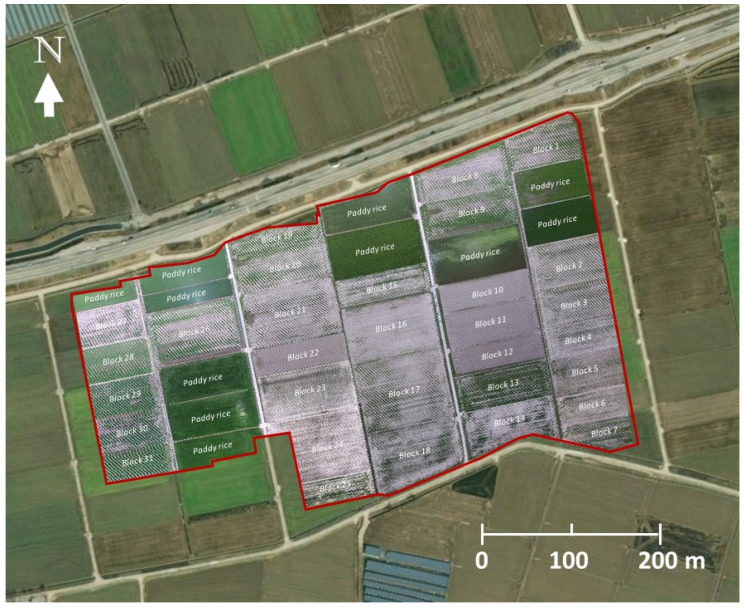
A composite image (outlined in red) showing 31 soybean blocks and damage by *S. exigua*. The composite image is overlaid onto a base map in the geographic information system (GIS). See Table S1 for detailed information on the field size and amount of soybean damage.

**Figure 6:**
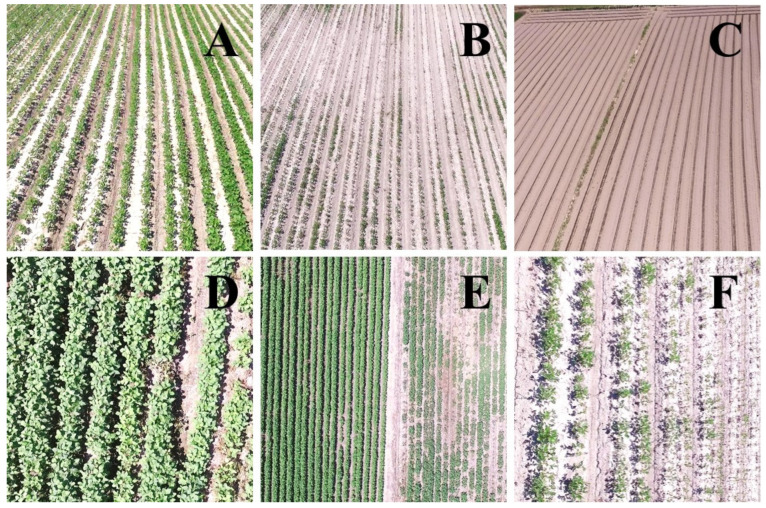
Example aerial photos of soybean fields taken by UAS. Typical soybean fields (**A**–**C**) with varying defoliation levels: (**A**), low defoliation; (**B**), high defoliation; (**C**), near 100% defoliation. Aerial views of healthy (**D**) and damaged (**F**) soybean fields and the two soybean blocks side by side with various levels of soybean defoliation by *S. exigua* (**E**).

A total of 150 aerial images were taken during the high-altitude UAS flight (Figure 3A). A low-altitude flight confirmed soybean defoliations in all 31 soybean blocks (Figure 4A). The composite image (Figure 5) showed various levels of soybean damage by *S. exigua*. Specifically, soybean blocks 10, 11, and 12 were completely defoliated by *S. exigua* (Figure 5 and Figure 6, Table S1).

**Figure 7:**
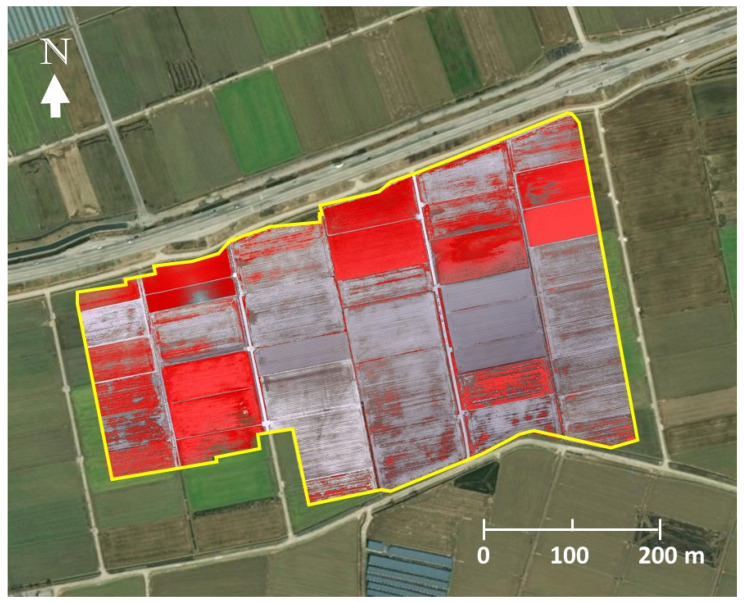
The finalized damage map by image analyses, which extracted foliage of soybean plants (red) in the study area.

The analysis of the aerial images showed that each pixel in the composite image corresponded to an area of 0.02 m^2^, roughly equivalent to one soybean plant. This allowed for estimation of plant-level defoliation. The results indicated that *S. exigua* feeding had removed 78.3% of soybean foliage in the study site; the total area of soybean fields planted was 10.11 ha, of which 7.92 ha were defoliated. The level of defoliation varied across soybean blocks, with the least and most severely affected blocks showing defoliation rates of 22.4% and 99.9%, respectively (Table S1 and Figure 7).

### 3.3. Economic Analysis to Determine the Efficiency of Aerial Survey

**Figure 8:**
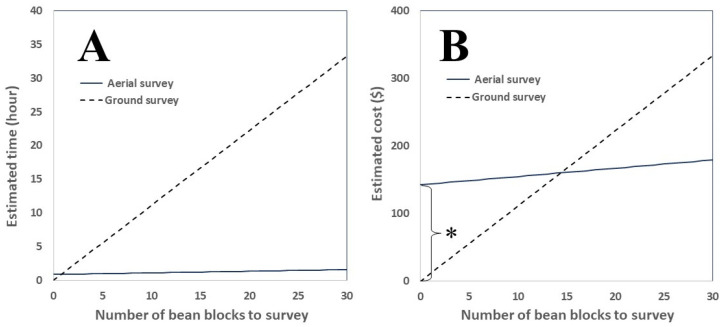
Comparison of estimated survey hour (**A**) and estimated cost (**B**) between aerial survey and ground survey. * indicates a technology fee including rental for the UAS and an image analysis tool.

The ground survey of 45 soybean blocks was completed by 19 surveyors in 1.1 h per surveyor (i.e., F_expert_ was set as 1.1). A pre-flight check, UAS inspection, and test flight were conducted before any UAS flight mission to ensure safe operation, taking 30 min. Both the high- and low-altitude aerial surveys took 57 min covering 20 ha. Therefore, the total time for UAS operation (T_UAS_) was 0.5 h for pre-flight check and 0.05 h per block. It took 2 h to finish image analysis for the 31 soybean blocks, and thus T_analysis_ was set as 0.0645 h per block. Using these parameter values, the economic analysis revealed that the aerial survey with UAS was more time efficient than the ground survey when more than one soybean block was surveyed (Figure 8A). In addition, the cost for the survey was a more economical aerial survey with UAS than that by ground surveys when more than 15 soybean blocks are surveyed (Figure 8B).

### 3.4. Spatial Patterns of Soybean Damage by S. exigua

The geostatistical analyses revealed that non-linear models, such as exponential, spherical, and Gaussian models, provided the best fit for the data, indicating spatial correlation of soybean damage caused by *S. exigua*. The degree of spatial dependence (*DD*) was greater than 75% in 10 of the 13 soybean blocks and 50–75% in 3 of the blocks (Table 1), indicating an overall strong spatial dependence of *S. exigua* damage. The range values of the semivariograms, which represent the maximum distance at which values are spatially correlated, ranged from 89 m to 872 m for the soybean blocks we surveyed.

SADIE results revealed that soybean damage by *S. exigua* was spatially aggregated or clustered; *I*_a_ was > 1 in all 13 blocks (Table 2). The aggregated distribution of soybean damage was statistically significant in 12 blocks (*P*_a_ < 0.05). Strong clustering into gaps and patches ($\overset{-}{v_{i}}$ > 1.5 and $\overset{-}{v_{j}}$ < −1.5) verified significant spatial aggregation of soybean damage in the 12 soybean blocks (*P*$\overset{-}{v_{i}}$ < 0.05 and *P*$\overset{-}{v_{j}}$ < 0.05).

### 3.5. Regional-Scale Soybean Damage by S. exigua

**Figure 9:**
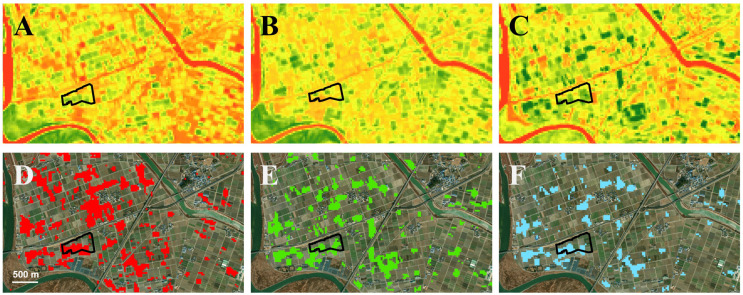
NDVI values (darker green indicates healthier vegetation, and darker red indicates unhealthier vegetation) before (**A**), during (**B**), and after (**C**) the *S. exigua* outbreak. Areas with red color in (**D**) indicate soybean fields with *S. exigua* damage calculated from the difference in NDVI values between (**A**,**B**). Areas with green color (**E**) indicate soybean fields with recovery, regrown, or replanted after the *S. exigua* outbreak, which was calculated from the difference in NDVI values between (**B**,**C**). Areas with blue color (**F**) show the overlapping areas of (**D**,**E**), indicating the areas were damaged by *S. exigua* but also recovered or replated after the damage. An area with a black outline is the site for this study.

The NDVI values before (Figure 9A), during (Figure 9B), and after (Figure 9C) the *S. exigua* outbreak exhibited significant changes. The differences in NDVI values between before and during the *S. exigua* outbreak ranged from 0.058 to 0.683 (Figure 9D), indicating vegetation losses resulting from defoliation. Similarly, the NDVI values between during and after the *S. exigua* outbreak ranged from 0.066 to 0.582, corresponding to fields where soybeans had been replanted or had regrown following the *S. exigua* outbreak (Figure 9E,F). Within our study sites (outlined with a black polygon in Figure 9), 20 out of 30 soybean blocks were identified as damaged or exhibiting regrowth/replanting based on the NDVI values, while the defoliation in the other 10 blocks was not detected through the changes in NDVI values before, during, and after the *S. exigua* outbreak (Table S2).

## 4. Discussion and Conclusions

UAS have been increasingly utilized for various agricultural applications, such as crop and soil monitoring [31,32,33,34,35], pest detection [3,36], aerial releases of natural enemies [7,37], and pesticide application [38]. With the availability of small rotary-wing UAS equipped with high-resolution cameras, it is now possible to detect insects directly from aerial images [34]. Such high image resolution was also observed in our study (see Figure 1B for visible feeding signs on a soybean leaf). This level of resolution could be utilized to detect soybean damage caused by *S. exigua* at later crop stages, such as the flowering stage, even with lower levels of defoliation. However, remote sensing with UAS and precision agriculture are data-intensive procedures that often require skilled personnel [39]. In addition, automated detection of insect damage through image analysis is needed to make this technology more accessible to general agricultural practitioners. Our study demonstrated large-scale detection and rapid assessment of *S. exigua* damage using UAS.

Another technology that can be used for the detection of the *S. exigua* outbreak is satellites. Given the extensive damage to soybeans caused by the *S. exigua* outbreak, time series data containing spectral change information obtained from satellite imagery can be utilized to map insect damage at the regional level [40]. Our post-hoc analysis showed that the differences in NDVI values between before and during the *S. exigua* outbreak indicated vegetation losses due to defoliation. Similarly, the NDVI values between during and after the *S. exigua* outbreak coincided with fields where soybeans had been replanted or regrown after the *S. exigua* outbreak (Figure 9E). These results suggest that satellite data can be used to identify the hotspots of soybean defoliation in a large area with high levels of damage. However, it is still difficult to precisely locate individual soybean blocks with defoliation by *S. exigua* by using satellite and the changes in NDVI values before, during, and after the outbreak. In addition, the quality of Landsat 8 images is dependent on weather conditions, and the resolution is moderate when calculating NDVI using a moderate resolution imaging spectroradiometer (MODIS) sensor on satellites. As a result, obtaining suitable images for calculating NDVI values during a short pest outbreak period can be challenging. Our study is the first to detect the outbreak of *S. exigua* using NDVI, although a few previous studies have successfully demonstrated the potential of satellite-based NDVI in detecting outbreaks of the fall armyworm *S. frugiperda* in cornfields [40,41].

The results of this study indicate that aerial surveys using small rotary-wing UAS offer several advantages for detecting and managing insect outbreaks. One of the major advantages is that aerial surveys with UAS are often more cost-effective compared to conventional ground-based surveys. In the case of the *S. exigua* outbreak in South Korea, our study demonstrated that using UAS was more economical than conducting a ground survey when the number of target soybean blocks exceeded 15. This is because the UAS can cover a larger area in a shorter amount of time and with fewer personnel, thereby reducing survey costs. Another advantage of aerial surveys is that it can provide valuable information in the form of maps that show the distribution of insect damage. These maps can be used to guide where to conduct intensive surveys and management in the outbreak area, as well as to inventory the areas of damage. A digital inventory of insect outbreaks can be a useful resource for predicting and preventing future outbreaks, especially in the case of pests such as *S. exigua*, which have a history of occurring and damaging a wide variety of high-value crops periodically in South Korea since the first outbreak was reported in 1926 [42].

Site-specific management of *S. exigua* is another benefit of using UAS for outbreak surveys. By identifying the locations of damage, management measures can be applied only where needed [43], reducing pesticide inputs and increasing control efficiency [43,44]. Our study revealed that soybean damage caused by *S. exigua* exhibited spatial aggregation, and a previous study [16] also demonstrated the spatial aggregation of the larval stage of *S. exigua*. Therefore, UAS can collect detailed spatial data through aerial images, enabling the identification of hotspots and the spatial distribution of pest damage. This information can contribute to precise pest management practices and a reduction in sampling costs.

Additionally, our study found that the amount of defoliation by *S. exigua* varied considerably between adjacent soybean blocks (Figure 6C–E), suggesting that changing soybean variety or planting time could help avoid or reduce damage. This study showed that a major cost of using a small UAS for insect outbreak surveys was fees for the use of technology, including UAS and image analysis. As the price of such technology has decreased with technological advances [7] the use of UAS and image analysis could be cheaper in the future.

Finally, advances in technology have made UAS and image analysis more affordable, as well as the use of learning-based methods [45]. More recently developed convolutional neural networks [46,47] could further improve automated image processing and outbreak detection capabilities in the future. Artificial intelligence and machine learning would allow for more accurate and efficient monitoring and management of insect outbreaks. Specifically, deep learning in artificial intelligence (e.g., Mask2former model) is one of the potential tools for visual recognition of target pests [6], which could be used for early detection of insect pest outbreaks.

In conclusion, our study provided clear evidence of the effectiveness of utilizing an autonomous drone and image analysis for conducting a cost-effective aerial survey of soybean damage caused by *S. exigua* during an outbreak. The integration of UAS and image analysis proved to be an efficient and economical approach for assessing the extent of soybean damage caused by *S. exigua*. By swiftly assessing the magnitude of outbreaks and identifying areas requiring urgent management, rapid responses to further damage can be achieved.

Furthermore, our study involving UAS and satellite data demonstrated that precise assessments of pest outbreaks can facilitate the development of targeted pest management strategies, such as site-specific pest management [43]. The utilization of remote sensing through UAS and sensor technologies has become an essential method for site-specific pest management, as evidenced in our study. We discovered significant spatial aggregation of soybean damage caused by *S. exigua* and observed spatial autocorrelation across a wide area, highlighting the importance of employing this approach for effective pest management interventions.

## Figures and Tables

**Table 1:** Geostatistical description of soybean damage by *S. exigua*. Note that soybean blocks not shown in this table had >95% damage, so their pattern was not analyzed.

Block	Model	Nugget	Sill	*DD* (%)	Range (m)	r^2^	RSS
Block 1	Exp.	14,400	212,600	93.2	89	0.92	7.53 × 10^8^
Block 7	Exp.	101,000	416,400	75.7	281	0.98	8.00 × 10^8^
Block 8	Sph.	91,000	494,100	98.1	606	0.98	3.22 × 10^9^
Block 9	Sph.	100	149,700	99.9	129	0.86	3.20 × 10^8^
Block 13	Sph.	15,930	38,320	58.4	272	0.95	1.08 × 10^7^
Block 18	Exp.	48,100	344,300	86.0	257	0.98	1.49 × 10^9^
Block 19	Gauss.	99,200	223,000	55.5	111	0.94	5.73 × 10^8^
Block 20	Exp.	41,000	401,000	89.8	166	0.93	4.59 × 10^9^
Block 25	Sph.	15,200	118,900	87.2	257	0.94	2.57 × 10^8^
Block 26	Exp.	194,000	560,400	65.4	872	0.97	1.09 × 10^9^
Block 28	Sph.	100	150,600	99.9	163	0.84	7.82 × 10^8^
Block 29	Sph.	100	149,700	99.9	129	0.86	3.20 × 10^8^
Block 30	Exp.	8100	53,550	84.9	137	0.98	2.39 × 10^7^
Block 31	Sph.	9910	25,110	60.5	752	0.87	1.72 × 10^7^

*DD*, degree of spatial dependence; RSS, residual sums of squares; Exp., exponential model; Sph., spherical model; Gauss., Gaussian model.

**Table 2:** SADIE parameters for the distribution soybean damage caused by *S. exigua* in each soybean block surveyed by UAS. Note that soybean blocks not shown in this table had >95% damage, so their pattern was not analyzed.

Block	*I*_a_	*P*_a_	$\overset{-}{v_{j}}$	$\overset{-}{v_{i}}$	$P\overset{-}{v_{j}}$	$P\overset{-}{v_{i}}$
Block 1	1.604	0.0095	−1.495	1.445	0.0118	0.0213
Block 7	1.846	0.0015	−1.883	1.788	0.0021	0.0036
Block 8	3.272	0.0003	−2.190	2.190	0.0000	0.0003
Block 9	2.132	0.0015	−1.582	1.548	0.0074	0.0131
Block 13	2.030	0.0008	−1.911	1.815	0.0010	0.0038
Block 18	5.742	0.0003	−1.878	2.135	0.0003	0.0000
Block 19	1.482	0.0633	−1.444	1.336	0.0679	0.1074
Block 20	1.518	0.0051	−1.659	1.563	0.0015	0.0038
Block 25	1.534	0.0279	−1.533	1.377	0.0318	0.0667
Block 26	2.736	0.0003	−1.910	1.883	0.0003	0.0003
Block 28	1.417	0.0287	−1.413	1.257	0.0287	0.0918
Block 29	2.132	0.0015	−1.582	1.548	0.0074	0.0131
Block 30	2.774	0.0003	−2.816	2.510	0.0000	0.0000
Block 31	1.702	0.0074	−1.610	1.503	0.0144	0.0290

*I*_a_ and *P*_a_, index of aggregation and associated *p* value; *v_j_* and *v_i_*, and *Pv_j_* and P*v_i_*, indices of clustering and associated *p* values.

## Data Availability

Data will be available upon request.

## Supplementary Materials

The following Supporting Information can be downloaded at: https://www.mdpi.com/article/10.3390/insects14060555/s1, Table S1: Damage of soybean by *S. exigua* in 31 blocks surveyed by UAS; Table S2: Comparison of change detection for NDVI values identified blocks and field damage.
