# Supplementary material for: Rapid Assessment of Insect Pest Outbreak Using Drones: A Case Study with Spodoptera exigua (Hübner) (Lepidoptera: Noctuidae) in Soybean Fields
Source: Insects. 2023 Jun 15;14(6):555. doi: 10.3390/insects14060555 (PMC10299355; doi:10.3390/insects14060555)
Supplement: Supplementary file 1 [file insects-14-00555-s001.zip › insects-2402972-supplementary.pdf]

Table S1. Damage of soybean by *S. exigua* in 31 blocks surveyed by UAS.

Soybean block	Area (m^2)	Damaged area (m^2)	Defoliation
Block 1	3,339	2,233	66.9%
Block 2	3,949	3,782	95.8%
Block 3	2,896	2,832	97.8%
Block 4	2,325	2,269	97.6%
Block 5	2,973	2,852	95.9%
Block 6	2,082	2,017	96.9%
Block 7	2,005	1,292	64.4%
Block 8	4,257	3,113	73.1%
Block 9	4,008	1,516	37.8%
Block 10	2,814	2,795	99.3%
Block 11	3,755	3,739	99.6%
Block 12	2,556	2,553	99.9%
Block 13	3,797	982	25.9%
Block 14	3,921	3,446	87.9%
Block 15	2,893	2,291	79.2%
Block 16	5,690	5,548	97.5%
Block 17	1,791	1,630	91.1%
Block 18	5,244	4,522	86.2%
Block 19	1,966	952	48.4%
Block 20	4,104	3,174	77.3%
Block 21	5,701	5,636	98.9%
Block 22	3,005	2,960	98.5%
Block 23	4,943	4,923	99.6%
Block 24	1,360	1,357	99.7%
Block 25	1,956	1,279	65.4%
Block 26	5,060	3,643	72.0%
Block 27	2,672	2,549	95.4%
Block 28	2,634	646	24.5%
Block 29	2,543	592	23.3%
Block 30	2,693	1,405	52.2%
Block 31	2,201	492	22.4%

Table S2. Number of soybean blocks with changes in NDVI values among before, during, and after the *S. exigua* outbreak, indicating soybean defoliation and recovery/replanting.

**Damage level**	**Number of soybean blocks**	**Number of been blocks where NDVI values descreased before and during *S. exigua* outbreak***	**Number of been blocks where NDVI values increased during and after *S. exigua* outbreak****
Severe	7	2	3
High	18	13	13
Medium	3	2	1
Low	3	3	3

* indicates defoliation of soybean by *S. exigua*, and ** indicates the regrowth or replanting of soybean after the *S. exigua* outbreak occurred.
